# Next-generation text-mining mediated generation of chemical response-specific gene sets for interpretation of gene expression data

**DOI:** 10.1186/1755-8794-6-2

**Published:** 2013-01-29

**Authors:** Kristina M Hettne, André Boorsma, Dorien A M van Dartel, Jelle J Goeman, Esther de Jong, Aldert H Piersma, Rob H Stierum, Jos C Kleinjans, Jan A Kors

**Affiliations:** 1 Department of Toxicogenomics, Maastricht University, Maastricht, The Netherlands; 2 Department of Medical Informatics, Erasmus University Medical Center, Rotterdam, The Netherlands; 3 Department of Human Genetics, Leiden University Medical Center, Leiden, The Netherlands; 4 Microbiology and Systems Biology, TNO, Zeist, The Netherlands; 5 Laboratory for Health Protection Research, National Institute for Public Health and the Environment (RIVM), Bilthoven, The Netherlands; 6 Human and Animal Physiology, Wageningen University, Wageningen, The Netherlands; 7 Department of Medical Statistics and Bioinformatics, Leiden University Medical Center, Leiden, The Netherlands; 8 Institute for Risk Assessment Sciences, Utrecht University, Utrecht, The Netherlands

**Keywords:** Text mining, Toxicogenomics, Gene set analysis

## Abstract

**Background:**

Availability of chemical response-specific lists of genes (gene sets) for pharmacological and/or toxic effect prediction for compounds is limited. We hypothesize that more gene sets can be created by next-generation text mining (next-gen TM), and that these can be used with gene set analysis (GSA) methods for chemical treatment identification, for pharmacological mechanism elucidation, and for comparing compound toxicity profiles.

**Methods:**

We created 30,211 chemical response-specific gene sets for human and mouse by next-gen TM, and derived 1,189 (human) and 588 (mouse) gene sets from the Comparative Toxicogenomics Database (CTD). We tested for significant differential expression (SDE) (false discovery rate -corrected *p*-values < 0.05) of the next-gen TM-derived gene sets and the CTD-derived gene sets in gene expression (GE) data sets of five chemicals (from experimental models). We tested for SDE of gene sets for six fibrates in a peroxisome proliferator-activated receptor alpha (PPARA) knock-out GE dataset and compared to results from the Connectivity Map. We tested for SDE of 319 next-gen TM-derived gene sets for environmental toxicants in three GE data sets of triazoles, and tested for SDE of 442 gene sets associated with embryonic structures. We compared the gene sets to triazole effects seen in the Whole Embryo Culture (WEC), and used principal component analysis (PCA) to discriminate triazoles from other chemicals.

**Results:**

Next-gen TM-derived gene sets matching the chemical treatment were significantly altered in three GE data sets, and the corresponding CTD-derived gene sets were significantly altered in five GE data sets. Six next-gen TM-derived and four CTD-derived fibrate gene sets were significantly altered in the PPARA knock-out GE dataset. None of the fibrate signatures in cMap scored significant against the PPARA GE signature. 33 environmental toxicant gene sets were significantly altered in the triazole GE data sets. 21 of these toxicants had a similar toxicity pattern as the triazoles. We confirmed embryotoxic effects, and discriminated triazoles from other chemicals.

**Conclusions:**

Gene set analysis with next-gen TM-derived chemical response-specific gene sets is a scalable method for identifying similarities in gene responses to other chemicals, from which one may infer potential mode of action and/or toxic effect.

## Background

In toxicogenomics, gene and protein activity within a particular cell or tissue of an organism in response to toxicants is studied with the aim to predict in vivo effects from in vitro models. The assumption that similar gene expression profiles dictate similar physiological responses underlies the use of gene expression profiling in toxicogenomics to discern the toxicological properties of a chemical entity. Connectivity mapping is a promising method, based on gene-expression similarity, that can be applied to toxicogenomic data to understand mechanisms of toxicity (for a recent review see [1]). In essence, toxicological properties of a chemical entity are discerned by connecting a query gene signature generated as a result of exposure of a biological system (whole animal, tissue or cell line) to the chemical, to other, already known, chemical entities via a database of chemical compound *reference* gene expression signatures (pioneered in [2]). In contrast to gene expression data *repositories* such as Chemical Effects in Biological Systems Knowledge Base (http://www.niehs.nih.gov/research/resources/databases/cebs/) that contains data from different laboratories running different experimental platforms on different biological samples, the data in a *reference* gene expression signature database comes from systematic screening of chemical compounds against specific cell lines simulating biological conditions. Unfortunately, building such a reference gene expression signature database with many different cell types and compound concentrations represented is not easily feasible due to high costs and long development time [3]. For example, in the case of the Connectivity Map (cMap) (http://www.broadinstitute.org/cmap/), which is the largest public reference gene expression signature database (7,000 expression profiles representing 1,309 compounds), not all small molecules were tested in every cell model, and not all were tested across the same spectrum of concentrations. Moreover, how to best interpret the result of a query is still an open question.

As an alternative to such reference databases of gene expression signatures, genes could be annotated to a chemical response through text mining (TM) techniques. Text mining is the use of automated methods for exploiting the enormous amount of information available in the biomedical literature. Next-generation text mining (next-gen TM) refers to knowledge discovery based on concept profile matching (see section "Next-gen TM for chemical-gene associations"), in comparison to first-generation text mining where associations are extracted purely from concept co-occurrence. The biomedical literature contains information about small molecules that is not currently stored in gene expression databases, and this information could be used to build chemical response-specific signatures. For example, searching the biomedical literature database PubMed (http://www.ncbi.nlm.nih.gov/pubmed/) with the CAS number and substance name search string “79983-71-4 OR hexaconazole” results in 69 entries, while no information about the chemical can be found in the CEBS Knowledge Base or cMap (search performed Nov 21, 2011). In addition, next-gen TM-derived chemical response-specific signatures would not be specific to a certain biological system or compound concentration, and might therefore provide a less biased view on compound action than standard connectivity maps. Given a gene expression experiment where a biological system has been exposed to a chemical, these next-gen TM-generated chemical response-specific signatures could then be tested against the gene expression data set using gene set analysis (GSA) methods [4] that utilize statistic tests suitable for this purpose. The test would produce a ranking of chemicals in a way similar to the results from a connectivity-mapping query, with the difference that the chemicals are represented by gene sets derived from the literature instead of gene expression signatures from a reference database. Gene set analysis has fast become one of the standard methods in bioinformatics, and there are many tools available (for a review of tools, see [5]). Most GSA tools provide gene sets based on the Gene Ontology (GO) [6], with only a few providing additional sources of gene sets such as metabolic pathways, protein domains, disease associations, tissue expression, transcription factors sequence motifs, miRNA sequences, drug-gene associations [5] and toxicologically relevant gene sets (Boorsma *et al.*, submitted). We hypothesize that more gene sets can be created by next-gen TM, and that these can be used with GSA methods for chemical treatment identification, for pharmacological mechanism elucidation, and for comparing compound toxicity profiles.

GSA with literature-derived chemical response-specific gene sets has been used before to relate chemical structures to gene expression patterns in microarray experiments. Minguez *et al.*[7] used their tool MarmiteScan to associate chemicals with the characteristics of acute myeloid leukemia cell differentiation. However, there is no information about the size and scope of the chemical dictionary they used to mine the literature and their gene sets are not separately available, thus forcing the researcher to use their GSA method. There is also no possibility to test a sub-set of the gene sets, for example only those that are relevant for evaluation of developmental toxicity. In contrast, we provide chemical response-specific gene sets that can be used with any GSA tool that allows for user-supplied gene sets. Patel and Butte [3] used the hypergeometric test to associate gene sets derived from curated chemical-gene interactions from > 4,000 chemicals in the Comparative Toxicogenomics Database (CTD) [8] with six gene expression data sets selected based on their diversity with respect to species, chemical exposure and cell type. Manual curation of chemical-gene interactions from publications is a very time-consuming process producing high-quality information but with limited coverage, reflected by the number of chemicals that Patel and Butte could create gene sets for (1338 chemicals). CTD's chemical dictionary is a modified subset of descriptors from the “Chemicals and Drugs” category and Supplementary Concept Records from the U.S. National Library of Medicine (NLM) Medical Subject Headings, a hierarchical vocabulary used to index PubMed articles containing > 100 000 chemicals. We aim to increase the number of chemicals that can be annotated with genes by using next-gen TM instead of manual curation. Jelier *et al.*[9] used the weighted global test, with weights based on drug-gene associations generated from next-gen TM, to associate known peroxisome proliferator-activated receptor alpha (PPARalpha) agonists with the gene expression differences between PPARalpha-null and wild-type mice exposed to the fibrate WY14643. Although Jelier *et al.* demonstrated the usefulness and performed extensive evaluation of the next-gen TM technology for gene set testing, its reliability still remains to be investigated by evaluating other chemical classes than drugs and considering a wider range of gene expression data sets using other GSA methods; this validation is one of the aims of the present study.

According to a predefined procedure which avoids researcher bias we generated gene sets using next-gen TM technology, and tested these gene sets using three different GSA tools: ToxProfiler (Boorsma *et al.*, submitted; http://ntc.voeding.tno.nl/toxprofiler_test/), which implements the unpaired *t*-test to score the difference in gene expression between the genes from a pre-defined gene set and the remainder of the genes, the weighted global test (http://www.bioconductor.org/packages/release/bioc/html/globaltest.html), which implements a regression model where the gene expression measurements correspond to the covariates and the phenotype corresponds to the response, and GeneCodis (http://genecodis.dacya.ucm.es), which implements the hypergeometric test for gene set over-representation in a list of differentially expressed genes with respect to a reference gene list. Hereby we would like to show that the usefulness of next-gen TM-based gene sets is not tied to a specific GSA method. To show the versatility of data for which the technology is applicable, we test our next-gen TM-based gene sets with the GSA tools in three different case studies. The first study focuses on identification of the chemical treatment, the second on pharmacological mechanism elucidation, and the third on compound toxicity profile comparison. We name these case studies 1, 2 and 3 in the rest of this paper.

Case study 1 comprises the same gene expression experiments used by Patel and Butte [3], as mentioned earlier. We aim to compare the performance of CTD-based gene sets with next-gen TM-based gene sets in predicting the particular chemical treatment response. In case study 2 we analyze the gene expression experiment used by Jelier and coworkers [9], as mentioned earlier. For this case study, we aim to predict the PPARalpha agonism characteristic of fibrates. Fibrates have a profound pharmacological response with well-defined molecular toxicological properties, and constitute a clear test case in which both methods (CTD, next-gen TM) together with different gene-set testing approaches (ToxProfiler, weighed global test, GeneCodis) should perform well. To compare our approach to connectivity mapping, we compare the results from the GSA tools with the results from the cMap. Since case study 2 is focused on associating drugs with a gene expression profile and cMap contains mainly drugs, this case study was considered an appropriate choice for which to include a comparison with cMap. In case study 3, we demonstrate the power of the next-gen TM-based gene sets to link chemicals with similar gene expression response, where the manually curated gene sets from the CTD fail due to lacking chemical-gene annotations. We do this by analyzing a recently published in vitro gene expression data set on three chemicals belonging to the triazole class of developmental toxicants [10] with the aim to find chemicals with known, or unknown (to our knowledge), links to the class. Using next-gen TM, we make chemical response-specific gene sets for the chemicals contained in the ToxRefDB_DevTox database (http://www.epa.gov/ncct/toxrefdb/) [11] and link these gene sets to the data set for the triazoles. For the same gene expression data set we show that next-gen TM-derived gene sets also can be used for other purposes than chemical similarity matching. As an example, gene sets associated with embryonic structures are used to discriminate triazoles from other developmental toxicants, and from non-developmental toxicants.

## Methods

### Associating chemicals with genes in the literature

#### Next-gen TM for chemical-gene associations

The next-gen TM-based gene sets were compiled based on a literature-derived matching score for the chemical-gene association. The technology uses the vector space model to relate two concepts (such as chemicals and genes) to each other [12]. Concepts originated from a thesaurus (see section “Thesaurus”) that contains terms referring to biomedical and chemical concepts, and a list of term synonyms. The vector space model yields a measure of the strength of the association through the matching score determined by the cosine of the angle between the two concept vectors [12]. We call the vectors “concept profiles”. More precisely, a concept profile is a list of concepts with for every concept a weight to indicate its association to the root concept, based on concept co-occurrence statistics from the scientific literature database PubMed (http://www.ncbi.nlm.nih.gov/pubmed). A total of 13,834,150 PubMed IDs from January 1, 1980 to January 6, 2011 were used to build the concept profiles (PubMed IDs referring to experiments used in the case studies in this work were excluded). The co-occurrence statistics were calculated as follows. Peregrine (https://trac.nbic.nl/data-mining/), a software that performs thesaurus-based indexing and disambiguation of concepts in text, was used together with the thesaurus to identify concepts in PubMed records. Stop words were removed and words were stemmed to their uninflected form by the lexical variant generator (LVG) normalizer (http://lexsrv3.nlm.nih.gov/LexSysGroup/Projects/lvg/current/docs/userDoc/tools/norm.html). For all concepts except genes the PubMed records were comprised of the texts in which the concept was mentioned (titles, abstracts and Medical Subject Headings). For genes only a subset of PubMed records were used in order to limit the impact of ambiguous terms and distant homologs. Gene Ontology (GO) terms are sometimes given as words or phrases that are infrequently found in the normal texts. To still provide broad coverage of GO terms, the PubMed records that were used as evidence for annotating genes with this GO term were added. For every concept in the thesaurus that was associated to at least five PubMed records, a concept profile was created. This concept profile is thus in reality a vector containing all concepts associated with the root concept by direct co-occurrence, weighted by the symmetric uncertainly coefficient [12]. The top 200 concepts in a concept profile were used when calculating the matching scores. To make the gene sets we used a concept profile matching score cut-off of 1e-04 [9] in combination with a maximum of 1000 gene associations per chemical. To allow for comparison between the CTD-based and next-gen TM-based gene sets, only chemicals with Chemical Abstract Service (CAS) numbers were included. Using these restrictions, we were able to create 30,211 chemical response-specific gene sets for mouse and human.

#### Thesaurus creation

The thesaurus was composed of four parts: the 2010AB version of Unified Medical Language System (UMLS) (http://www.nlm.nih.gov/research/umls/), a gene thesaurus derived from multiple databases [13]), a chemical thesaurus derived from multiple databases [14], and a toxicology thesaurus derived from the International Union of Pure and Applied Chemistry (IUPAC) glossary of terms used in toxicology (http://sis.nlm.nih.gov/enviro/iupacglossary/frontmatter.html). For each part, we gather the synonyms and the definition for each concept. We then execute a number of rewriting and suppression rules based on term structure [13,14], and perform a manual analysis step of the top 250 frequent terms from a PubMed-indexation using Peregrine. Next, terms in the thesaurus are checked for in-thesaurus homonyms, and the 250 terms with the most homonyms are inspected manually for removal. When forming the master thesaurus, the UMLS, gene, chemical and toxicity thesauri are merged based on term overlap and patterns for recognizing gene and protein names [13]. The different steps are performed by a series of coupled java scripts.

#### Processing the CTD for chemical-gene associations

The CTD includes manually curated cross-species interactions between chemicals, genes, and diseases. CTD specifically targets individual chemicals for curation from a priority list; in addition, CTD also incidentally curates all interactions appearing in individual articles irrespective of whether they happen to have been targeted. We downloaded the chemical–gene interaction database from the CTD on January 6, 2011. The database contained 266,266 interactions in total. After filtering for *H. sapiens* and *M. musculus* (the two species included in the gene expression experiments used in this paper), 185,792 interactions remained. Neither the nature nor the curation level of the chemicals were taken into account. All different gene interactions with a specific chemical were regarded as associations, and summarized into one chemical response-specific gene set. During this step, all interactions based on any of the gene expression experiments used in the case studies in this work were removed. From the single chemical-gene associations in the CTD, we created gene sets with at least five genes (similar to Patel and Butte [3]) per chemical. For every chemical-gene association, CTD provides the CAS number for the chemical (if available), the Entrez Gene ID for the gene, and the PubMed ID and organism for which the association was reported. When filtering for chemicals with a CAS number and restricting the organisms to human and mouse we were able to make a total of 1,189 (*H. sapiens*) and 588 (*M. musculus*) gene sets. Sometimes in the CTD, *H. sapiens* Entrez Gene IDs are incorrectly annotated to *M. musculus*. These *H. sapiens* Entrez Gene IDs were mapped to *M. musculus* Entrez Gene IDs using the Homologene database (http://www.ncbi.nlm.nih.gov/homologene).

### Tools selection

We selected three different GSA tools that all allow for user-supplied gene sets but implement different statistical tests. 1) ToxProfiler, which implements the unpaired *t*-test to score the difference in gene expression between the genes from a pre-defined gene set and the remainder of the genes [15]. 2) The weighted version of the global test [16], which implements a regression model where the gene expression measurements correspond to the covariates and the phenotype corresponds to the response. In the weighted global test the next-gen TM-derived matching scores are used to weigh the contribution of each gene in a gene set to the test [9]. 3) GeneCodis [17], where biological annotations, or relationships among annotations based on co-occurrence patterns, are tested for over-representation in a list of differentially expressed genes with respect to a reference gene list using the hypergeometric test or the chi-square test. ToxProfiler and the weighted global test do not require a pre-selection of differentially expressed genes while GeneCodis does require such a list. For all tools, *p*-values were corrected for multiple testing using the False Discovery Rate (FDR) [18], and corrected *p*-values < 0.05 were considered significant.

In case study 2 we use the text-mining tool Anni (http://www.biosemantics.org/anni) [12] to explore the relevance of the top significant results on a biological process level. Anni is based on the same concept profile technology that was used to generate the next-gen TM-based gene sets, and provides direct links to the literature for the produced annotations.

### Gene expression microarray data sets selection and pre-processing

#### Gene expression profiling of Bisphenol A effects on human Ishikawa cells (GEO accession number: GSE17624, set short name: BPA)

The aim of the BPA study was to provide a comprehensive evaluation of changes in gene expression during treatment with Bisphenol A in vitro, and the study was performed using five doses at three different time points with four replicates each [19]. RNA was hybridized on an Affymetrix Human Genome U133 Plus 2.0 Array. For this study, we used the high dosed (10 nM) cells at 48 hours (four treated samples and four control samples).

#### Gene expression profiling of 17 beta-Estradiol effects on human MCF7 breast cancer cells (GEO accession number: GSE11352, set short name: ESThsa)

The aim of the ESThsa study was to identify 17 beta-Estradiol responsive genes in the estrogen-receptor positive breast cancer cell line, MCF7 [20]. MCF7 cells were exposed to 10 nM Estradiol (or vehicle only) at 12, 24, and 48 hours. Each time point was performed in triplicate. RNA was hybridized on an Affymetrix Human Genome U133 Plus 2.0 Array. For this study, we used only the samples from 24 hours with their corresponding controls (three treated samples and three control samples).

#### Gene expression profiling of 17 beta-Estradiol effects on mouse thymus (GEO accession number: GSE2889, set short name: ESTmmu)

The aim of the ESTmmu study was to compare the effects of Estradiol and its analog Genistein on mouse thymus [21]. Control samples were from the mice that were untreated (day 0). Two treatments (Estradiol injection and Genistein diet) and three time points were studied. Two replicated samples at each time point and each treatment were collected. RNA was hybridized on an Affymetrix Mouse Expression 430A Array. For this study, we used only the samples from the mice treated with Estradiol (day 2) and their corresponding controls (two treated samples and two control samples).

#### Gene expression profiling of 2,3,7,8-tetrachlorodibenzo-p-dioxin (TCDD) effects on mouse liver (GEO accession number: GSE10082, set short name: TCDD)

The aim of the TCDD study was to map the complete spectrum of aryl hydrocarbon receptor (Ahr) dependent genes in male adult liver by contrasting mRNA profiles of Ahr-null mice (Ahr−/−) with those in mice with wild-type Ahr (Ahr+/+) [22]. Transcript profiles were determined both in untreated mice and in mice treated 19 h earlier with 1000 μg/kg TCDD. RNA was hybridized on an Affymetrix Mouse Genome 430 2.0 Array. For this study, we used only the data from the wild-type mice (six treated samples and five control samples).

#### Gene expression profiling of 1alpha,25-Dihydroxyvitamin D3 (VitD3) effects on bronchial smooth muscle cells (GEO accession number: GSE5145, set shortname: VitD3)

The aim of the VitD3 study was to study gene regulation in bronchial smooth muscle cells following VitD3 stimulation [23]. The cells were from the same patient in all hybridization. Cells were treated for 24 hours with 100 nM of VitD3 or with the same concentration of vehicle (ethanol at 0.05%). The experiment was done in triplicates and a total of six samples (three treated and three control) were analyzed. RNA was hybridized on an Affymetrix Human Genome U133 Plus 2.0 Array.

#### Gene expression profiling of zinc sulfate (ZnSO4) effects on human bronchial epithelial cells (GEO accession number: GSE2111, set short name: ZnSO4)

The aim of the ZnSO4 study was to discriminate vanadium (VOSO4) from ZnSO4 using gene profiling [24]. Human bronchial epithelial cells were treated with vehicle (control), VOSO4 (50 uM) or ZnSO4 (50 uM) for four hours (four replicates each). RNA was hybridized on an Affymetrix Human Genome U133A Array. For this study, we used only the data from the four samples treated with ZnSO4 and their corresponding four control samples.

#### Gene expression profiling of WY14643 effects on mouse small intestine (GEO accession number: GSE9533, set short name: PPARA)

The aim of the PPARA study was to examine the effects of acute nutritional activation of PPARalpha on expression of genes encoding intestinal barrier proteins [25]. Male, four months old Wild-type (129S1/SvImJ) and PPARalpha −/− mice (129S4/SvJae) were exposed to dietary fatty acids with WY14643 as a reference during an exposure time of six hours, after which the small intestines were removed. RNA was hybridized on an Affymetrix Mouse Genome 430 2.0 Array. Here we use the samples corresponding to the PPARalpha activation by WY14643 (four treated samples and four control samples).

#### Gene expression data processing

The Affymetrix CEL files for all the different gene expression data sets listed above were downloaded from GEO and pre-processed using GenePattern [26]. CEL files were normalized applying Robust Multichip Average (RMA), using the ExpressionFileCreator module. In addition, the MBNI Custom CDF, which contains updated probe set definitions for Entrez GeneIDs, was applied: http://brainarray.mbni.med.umich.edu/Brainarray/Database/CustomCDF/11.0.1/entrezg.asp. After normalization, the data was floored by setting a threshold value of 50 for all probe sets. Log(2)ratios were created by dividing the median of the treatment values of each probe set by the median of the control values. Probe sets were discarded if both values were equal to 50.

No supplementary CEL files were available for the zinc sulfate dataset (GEO: *GSE2111*) and the Estradiol mouse dataset (GEO: *GSE2889),* instead the provided MAS5-calculated signal intensities were used to calculate the log(2)ratios. Affymetrix probeset identifiers were mapped to entrez gene identifiers using the appropriate affymetrix CDF. Log(2)ratios were created by dividing the median of the values of each probe set by the median of the time matched control where only probesets were selected that contained a “present” flag.

#### Gene expression profiling of triazole effects on mouse embryonic stem cell differentiation (ArrayExpress accession number: E-MTAB-300, set short name: triazoles)

In the triazoles experiment, *M. musculus* embryonic stem cells were exposed to a range of developmental toxicants and non-developmental toxicants (carbamazepine, flusilazole, hexaconazole, methotrexate, methylmercury chloride, monobutyl phthalate, monoethylhexyl phthalate, monomethyl phthalate, nitrofen, saccharine, triadimefon, warfarin) with the aim to evaluate developmental toxicant identification using gene expression profiling in embryonic stem cell test (EST) differentiation cultures [10]. RNA was hybridized on an Affymetrix Mouse Genome 430 2.0 Array. For the analysis with the weighted global test, we used the data at the 24 hour timepoint for the three different triazoles (flusilazole, hexaconazole, and triadimefon), one negative control (saccharine), and one unexposed control (dimethyl sulfoxide), each with eight replicates. The Affymetrix CEL files for the triazoles gene expression data set were normalized using the expresso package in R with the default settings. Due to the large number of replicates, no probe filtering was performed. Probesets were annotated with Entrez gene IDs using the bioconductor 4302.db package. To summarize the data at the Entrez gene ID level, read-outs from probesets with the same Entrez gene ID were averaged. For the PCA analysis, we used the data from all groups. The Affymetrix CEL files were normalized using Robust Multichip Average (RMA) normalization and probe to gene mapping was performed as described previously [27]. Probe sets for Affymetrix internal controls or probe sets that did not correspond to an Entrez gene ID were not used in further analyses.

## Results

### Case study 1

In this case study we aimed to compare the performance of CTD-based gene sets with next-gen TM-based gene sets in predicting the particular treatment response for six gene expression data sets. The *H. sapiens* gene sets were tested with the GSA tools against the human gene expression data sets (BPA, ESThsa, VitD3 and ZnSO4) and the *M. musculus* gene sets were tested with the GSA tools against the mouse gene expression data sets (ESTmmu and TCDD). Ranking based on the FDR-corrected *p*-value for the gene sets from each method (CTD, next-gen TM) for each GSA tool was used as outcome measure. The CTD-based gene sets ranked consistently and considerably higher than the next-gen TM-based gene sets over all GSA tools, with one exception: the next-gen TM-based ZnSO4 gene set ranked higher on the ZnSO4 gene expression data set using ToxProfiler (Table 1). On average, the gene set representing the treatment was significantly altered in three experiments using the next-gen TM-based gene sets and in five experiments using the CTD-based gene sets (Table 1). An exception is the weighted global test, for which both approaches scored significant in five experiments.

**Table 1:** Chemical treatment prediction ranks

	**ToxProfiler**		**Weighted global test**		**GeneCodis single annotation**	
	**CTD**	**TM**	**CTD**	**TM**	**CTD**	**TM**
BPA	**9 (2.0e-07)**	92 (1.0e-01)	**18 (3.9e-04)**	**21 (6.2e-05)**	**10 (9.1e-08)**	-
ESThsa	38 (8.8e-02)	400 (2.4e-01)	**269 (3.2e-03)**	**920 (5.6e-04)**	**4 (2.4e-02)**	-
ESTmmu	**18 (3.5e-05)**	**189 (1.3e-04)**	360 (5.7e-01)	482 (4.3e-01)	-	-
TCDD	**19 (8.0e-07)**	**89 (1.2e-03)**	**14 (2.9e-10)**	**84 (5.7e-10)**	**1 (5.3e-32)**	**89 (1.7e-05)**
VitD3	**9 (4.5e-02)**	400 (3.0e-01)	**186 (2.4e-03)**	**762 (6.0e-04)**	**1 (2.0e-26)**	**200 (6.0e-03)**
ZnSO4	**9 (3.8e-05)**	**6 (3.4e-04)**	**3 (2.6e-06)**	**4 (6.8e-05)**	-	-

Chemical treatment prediction rank and false discovery rate-corrected *p*-value (within parentheses) per gene expression data set for the different gene set analysis tools. Significant results (false discovery rate-corrected *p*<0.05) are in bold. GeneCodis only reports results with a false discovery rate-corrected *p*-value smaller than 0.05. Missing values denote such non-significant results.

**Figure 1:**
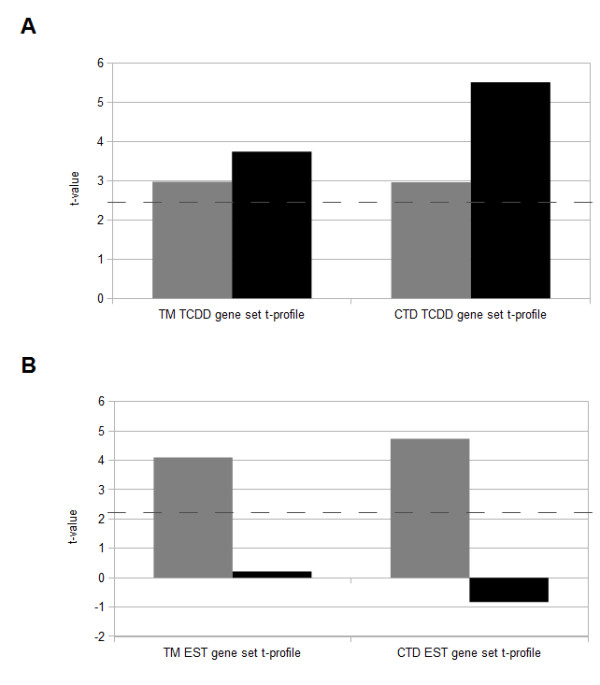
**T-profiles of mouse gene sets. **T-profiles of the CTD-based and next-gen TM-based TCDD mouse gene sets (**A**) and Estradiol (EST) mouse gene sets (**B**) for the TCDD (black bar) and ESTmmu (dark gray bar) mouse gene expression data sets. The dotted line represents the border of significant t-values.

**Figure 2:**
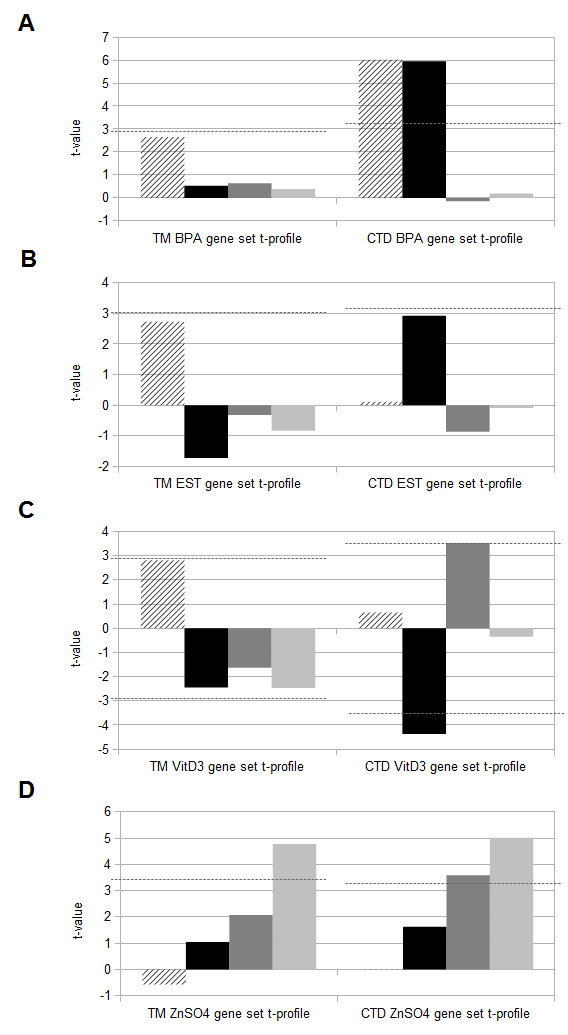
**T-profiles of human gene sets. **T-profiles of the CTD-based and next-gen TM-based BPA human gene sets (**A**), the Estradiol (EST) human gene sets (**B**), the VitD3 human gene sets (**C**), and the ZnSO4 human gene sets (**D**) for the BPA (striped bar), ESThsa (black bar), VitD3 (dark gray bar) and ZnSO4 (light gray bar) human gene expression data sets. The dotted line represents the border of significant t-values.

The statistical test behind ToxProfiler allows for comparison of gene sets over different gene expression data sets based on the t-values produced by the test [15]. Such a profile for a gene set is called a t-profile, and gives information about the specificity of the gene set (that is, if the gene is significantly differential expressed in more than one data set). We compared the t-profiles for the CTD-based and next-gen TM-based gene sets that corresponded to the chemical treatments for the data sets. The t-profiles were made on species level so that the gene sets containing mouse genes were compared across the mouse gene expression data sets and the gene sets containing human genes were compared across the human gene expression data sets. T-profiling of the CTD-based and next-gen TM-based gene sets corresponding to the chemical treatment for the different gene expression data sets showed similar t-profiles for three (Figure 1A and B, and Figure 2D) of the six gene sets. The other three gene sets (Figure 2A-C) had a dissimilar t-profile.

The CTD-based gene sets ranked highest when using the GeneCodis single annotation option (Table 1), but for some data sets the gene set representing the treatment did not score significant. For CTD, the Estradiol and ZnSO4 treatments were not significant. When opting for co-occurring annotations, GeneCodis additionally predicted the CTD-based Estradiol mouse gene set for the ESTmmu gene expression data set as significant with a rank of 14, together with the co-occurring gene sets TCDD and Tretinoin. Zinc and zinc chloride CTD-based gene sets were reported as significant at rank 1 together for the ZnSO4 gene expression data set, but not ZnSO4. For next-gen TM, three additional gene sets were reported significant when opting for co-occurring annotations: the Estradiol human gene set for the ESThsa gene expression data set with a rank of 164 together with nine other chemicals; the Estradiol mouse gene set for the ESTmmu gene expression data set with a rank of 41 in a cluster with 11 other chemicals; the ZnSO4 gene set for the ZnSO4 gene expression data set with a rank of 1 in a cluster together with 12 other chemicals.

Among the other tools, the weighted global test had the highest number of significant scoring gene sets while ToxProfiler had the better ranking in most cases (Table 1).

In short, the CTD-based gene sets ranked higher than the next-gen TM-based gene sets for all gene expression data sets except the ZnSO4 data set, but t-profiling indicated a similar significance pattern for 50% of the next-gen TM-based and CTD-based gene sets.

### Case study 2

In this case study we analyzed a PPARalpha-knock-out gene expression data set with the aim to predict the PPARalpha agonism characteristic of fibrates. When intersecting the CAS numbers for the CTD and next-gen TM gene sets, 585 *M. musculus* gene sets remained. The resulting 585 *M. musculus* CTD-based and next-gen TM-based gene sets were tested with the GSA tools against the PPARA gene expression data set, and compared to the results from the cMap. As mentioned before, ToxProfiler and the weighted global test do not require a list of differentially expressed genes, but GeneCodis does. To generate the list of differentially expressed genes needed for the analysis with GeneCodis, we used the topTable function from the bioconductor Limma package in R to obtain a ranked list of probes with the most evidence of differential expression between the knockout and wild-type samples. Probes with an adjusted *p*-value of less than 0.05 where kept (930 probes). Probesets were annotated with EntrezGene IDs using the bioconductor 4302.db package. To compare the results with cMap, we started with the same probesets and proceeded to make the query signature. cMap only accepts probes from the Affymetrix GeneChip Human Genome U133A Array. We therefore used the NetAffx tool supplied by Affymetrix (http://www.affymetrix.com/analysis/netaffx/index.affx) to map the probe IDs from our signature to Affymetrix GeneChip Human Genome U133A Array IDs.

Fibrate gene sets ranking and number of relevant biological processes annotated to the significant scoring chemical response-specific gene sets were used as outcome measures. All six next-gen TM-based fibrate gene sets ranked among the top-10 significant results in ToxProfiler and GeneCodis (using the single annotation option in GeneCodis) (Table 2). Three CTD-based fibrate gene sets ranked within the top-10 significant results in ToxProfiler and GeneCodis (using the single annotation option in GeneCodis) (Table 2). When opting for co-occurring annotations in GeneCodis, GeneCodis additionally predicted the CTD-based ciprofibrate gene set as significant with a rank of 554 in a cluster together with Acetaminophen, WY14643, TCDD, and Ethinyl Estradiol. All six fibrate gene sets scored significant for both CTD and next-gen TM using the weighted global test, but with lower average rank than for the other tools (Table 2). Jelier and co-workers [9] used the weighted global test to test four of the fibrates and reported ranks between 210 and 378. However, they used a different selection of chemicals (the semantic category "drugs" in the thesaurus) to test against the gene expression experiment. When testing the same selection of chemicals but with our concept profile matching association scores, the fibrates ranked between 183 and 304.

**Table 2:** Fibrate drug prediction ranks

	**ToxProfiler**		**Weighted global test**		**GeneCodis single annotation**	
	**CTD**	**TM**	**CTD**	**TM**	**CTD**	**TM**
clofibrate	**7 (6.1e-10)**	**2 (<1e.-10)**	**22 (1.0e-03)**	**71 (1.0e-06)**	**5 (1.1e-04)**	**2 (7.6e-29)**
gemfibrozil	53 (2.0e-01)	**8 (<1e.-10)**	**35 (2.0e-03)**	**106 (5.4e-06)**	-	**6 (3.5e-19)**
bezafibrate	54 (2.5e-01)	**7 (<1e.-10)**	**42 (2.1e-03)**	**105 (5.3e-06)**	-	**5 (9.3e-22)**
fenofibrate	**3 (<1e.-10)**	**10 (<1e.-10)**	**27 (1.6e-03)**	**115 (7.1e-06)**	**3 (2.2e-07)**	**9 (1.5e-18)**
ciprofibrate	**33 (4.2e-02)**	**5 (<1e.-10)**	**21 (8.7e-04)**	**97 (2.6e-06)**	**-**	**3 (3.1e-25)**
WY14643	**2 (<1e.-10)**	**4 (<1e.-10)**	**12 (5.5e-05)**	**118 (7.2e-06)**	**1 (3.2e-79)**	**4 (6.5e-22)**

Fibrate drug prediction ranks and false discovery rate-corrected *p*-values (within parentheses) for the PPARA gene expression data set for the different gene set analysis tools. Significant results (false discovery rate-corrected *p*<0.05) are in bold. GeneCodis only reports results with a false discovery rate-corrected *p*-value smaller than 0.05. Missing values denote such non-significant results. ToxProfiler does not report false discovery rate-corrected *p*-value less than 1.e-10. These are denoted as <1.e-10 in the table.

None of the fibrate signatures in cMap scored significant against the PPARA gene expression set signature.

**Figure 3:**
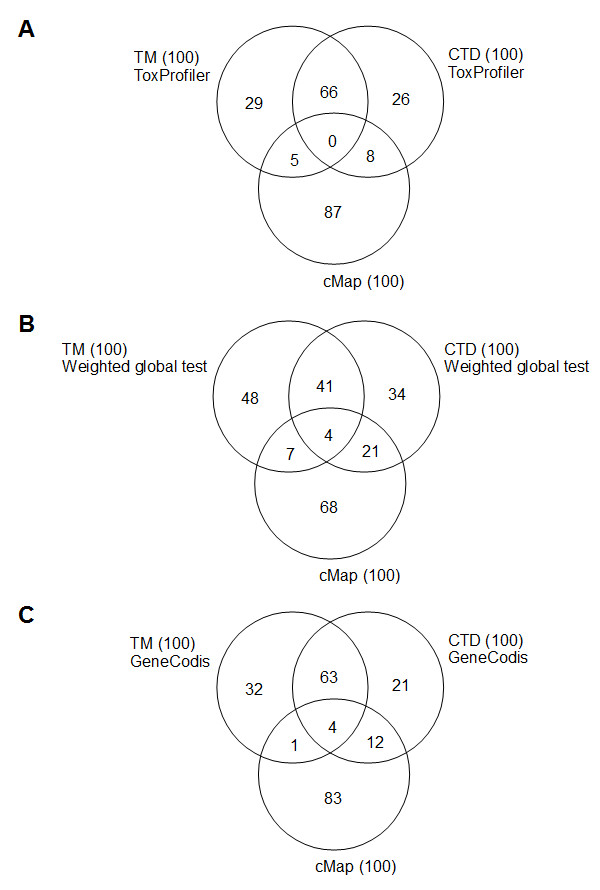
**Venn diagrams showing the overlap in biological processes. **Venn diagrams showing the overlap in biological processes for the most significant (false discovery rate-corrected *p*-value<0.05) chemicals between ToxProfiler and cMap (**A**), between the weighted global test and cMap (**B**), and between GeneCodis and cMap (**C**).

In the PPARA study [25], a list of differentially expressed genes was manually annotated with the following categories of biological processes: fatty acid oxidation, cholesterol flux, glucose transport, amino acid metabolism, intestinal motility, and oxidative stress. To investigate if the significant chemicals from the GSA tools and cMap were annotated with these biological processes, we matched the concept profiles for the significantly scoring chemicals against the concept profiles of the following semantic categories in Anni: cell function, molecular function, molecular dysfunction, organ or tissue function (this combination of semantic categories covered all biological process categories from the PPARA study). If more than 100 chemicals had scored significant using the GSA tools and cMap, the top-100 were used for the analysis. Venn diagrams of overlapping top-100 biological processes for the significant chemicals for each GSA tool and cMap showed higher overlap between CTD and next-gen TM, than between cMap and CTD, or cMap and next-gen TM (Figure 3). For ToxProfiler and cMap, no common concept could be found for CTD, next-gen TM and cMap, but 13 matched either next-gen TM or CTD (Figure 3A). For the weighted global test and cMap, four common concepts were found (Figure 3B), as for GeneCodis and cMap (Figure 3C). We then manually inspected the top 100 biological processes. Among the biological processes that were manually annotated to a list of differentially expressed genes in the PPARA study, fatty acid oxidation, cholesterol flux, glucose transport and oxidative stress were found among the top-100 biological processes for the significant chemicals for all GSA tools. For the significant chemicals from cMap, fatty acid oxidation and intestinal motility concepts where found.

In short, the next-gen TM-based fibrate gene sets ranked similar to or better than the CTD-based fibrate gene sets using two of the GSA tools, and all next-gen TM-based fibrate gene sets were significant using all GSA tools. The top-scoring chemicals from the GSA analyses represented underlying biological processes relevant to the gene expression experiment, both for the CTD-based and next-gen TM-based gene sets. In contrast, none of the fibrate signatures in cMap scored significant against the PPARA gene expression set signature, and fewer relevant biological processes were found for the top-ranking chemicals from cMap.

### Case study 3

The triazoles gene expression data set was analyzed with two aims: 1) to link chemicals with biological effects derived from -omics data similar to triazoles, and 2) to discriminate triazoles from other developmental toxicants (carbamazepine, methotrexate, methylmercury chloride, monobutyl phthalate, monoethylhexyl phthalate, nitrofen, warfarin), and from non-developmental toxicants (monomethyl phthalate and saccharine) using gene sets associated with embryonic structures.

Only the weighted global test was applied in this case study. The triazoles gene expression data set show very small variation in gene expression levels [10]. The weighted global test has a very strong null hypothesis, asserting that no gene with a positive importance weight is associated with the response, making it an appropriate test for analyzing such a data set.

For aim 1, a subset of the 30,211 next-gen TM gene sets derived from chemicals with CAS numbers was used. We only considered mouse genes, since the gene expression experiment was performed on mouse embryonic stem cells. Chemicals were selected based on the list of 384 compounds tested for developmental toxicity contained in the ToxRefDB_DevTox database. The number of similar morphological developmental toxicity endpoints in vivo in rabbit or rat (as recorded in the ToxRefDB_DevTox database) between the significantly scoring chemicals and the triazoles was used as outcome similarity measure. The morphological developmental toxicity endpoints were the following: cleft lip and/or cleft palate; variations or abnormalities of the limb, including scapula, clavical and pelvis; variations or abnormalities of the vertebral column, ribs or sternum; variations or abnormalities of the cranium; abnormalities of the metanephric kidney; and abnormalities of the ureter. The triazoles also caused general developmental toxicity endpoints: change in weight of fetus at near-term of pregnancy; histopathological, clinical, or unclassified abnormalities in fetus; preimplantation loss, postimplantation loss (resorptions) or fetal death impacting litter size; and pregnancy loss or maternal wastage, but these endpoints were not considered specific enough to be included in the similarity outcome measure.

After matching the CAS numbers of the chemicals in the ToxRefDB_DevTox to the CAS numbers of compounds with next-gen TM-based gene sets, 319 gene sets remained. Matching the CAS numbers of the chemicals in the ToxRefDB_DevTox to the CAS numbers for the CTD gene sets resulted in 30 gene sets. Of the triazoles used in this study, only triadimefon was included in the CTD gene sets. All triazoles used in this study were included in the next-gen TM gene sets (see http://www.biosemantics.org/index.php?page=chemical-response-specific-gene-sets), including Hexaconazole for which no information could be found in the CTD (as stated in the Background section). Therefore, we decided to continue the analysis using only the next-gen TM-based gene sets. Testing the 319 gene sets against the triazoles gene expression data set with the weighted global test resulted in 33 chemicals with significant changes in gene expression compared to untreated controls (Table 3). Of these 33 chemicals, 21 had a similar morphological developmental in vivo toxicity pattern as the triazoles according to the ToxRefDB_DevTox (at least one similar morphological developmental toxicity endpoint (see section Materials and Methods). Six of these were triazoles: diniconazole, difenoconazole, febuconazole, triadimenol, myclobutanil, and cyproconazole. Of the remaining 12 significantly scoring chemicals, nine had a non-specific pattern of in vivo toxicity compared to the triazoles according to the ToxRefDB_DevTox database (at least one similar general developmental toxicity endpoint), and three are reported as non-toxic in the ToxRefDB_DevTox database. Testing the 319 gene sets against the saccharine gene expression data set with the weighted global test resulted in five chemicals with significant changes in gene expression (Primisulfuron, Monosodium methane arsenate, Benfluralin, Pyridaben, Cyprodinil), of which four are developmentally toxic.

**Table 3:** Number of similar toxicological endpoints

**Chemical name**	**CAS number**	**# similar general developmental toxicity endpoints**	**# similar morphological developmental toxicity endpoints**	***p*-value**
**Metam-sodium**	137-42-8	3	3	5.6e-03
**Mepiquat chloride**	24307-26-4	0	2	9.3e-03
**Clomazone**	81777-89-1	2	3	9.3e-03
**6-Deisopropylatrazine**	1007-28-9	0	2	9.3e-03
Zoxamide	156052-68-5	0	0	9.3e-03
**Iprodione**	36734-19-7	4	2	9.3e-03
Monosodium methane arsenate	2163-80-6	3	0	9.3e-03
**Cyprodinil**	121552-61-2	2	2	9.5e-03
**Carfentrazone-ethyl**	128639-02-1	0	1	1.3e-02
**Primisulfuron-methyl**	86209-51-0	1	3	1.3e-02
3-(3,5-Dichlorophenyl)-1,5-dimethyl-3-azabicyclo(3.1.0)hexane-2,4-dione	32809-16-8	0	0	1.7e-02
Bromadiolone	28772-56-7	1	0	1.7e-02
MGK 264	113-48-4	0	0	2.5e-02
**Diniconazole**	83657-24-3	3	3	2.5e-02
**Difenoconazole**	119446-68-3	3	1	2.5e-02
Fenpropathrin	39515-41-8	1	0	2.5e-02
**Fenbuconazole**	114369-43-6	4	1	2.5e-02
Bendiocarb	22781-23-3	2	0	2.5e-02
Dacthal	1861-32-1	1	0	2.5e-02
**Fludioxonil**	131341-86-1	0	2	2.5e-02
**Mancozeb**	8018-01-7	3	3	2.5e-02
**Triadimenol**	55219-65-3	0	3	2.5e-02
**Myclobutanil**	88671-89-0	2	1	2.5e-02
**Cyproconazole**	94361-06-5	2	2	2.9e-02
Fluroxypyr	69377-81-7	1	0	3.1e-02
**Fluazifop-P-butyl**	79241-46-6	2	4	3.5e-02
**Ethametsulfuron methyl**	97780-06-8	4	2	3.5e-02
Cyclanilide	113136-77-9	0	0	3.8e-02
**EPTC**	759-94-4	4	1	4.1e-02
Metaldehyde	108-62-3	1	0	4.1e-02
Cyhexatin	13121-70-5	3	0	4.1e-02
**Pyrimethanil**	53112-28-0	3	2	4.1e-02
**Nitrapyrin**	1929-82-4	1	1	4.7e-02

Number of similar toxicological endpoints and false discovery rate-corrected *p*-values for chemicals predicted similar to the triazole gene expression data set using the weighted global test. Chemicals with a similar morphological developmental in vivo toxicity profile to triazoles are in bold.

For aim 2, we created a total of 442 gene sets associated with embryonic structures and tested these against the triazoles gene expression data sets with the weighted global test. The embryonic structure concepts originated from the semantic category "Embryonic Structure" from the UMLS part in our thesaurus, and the gene sets were created in a similar fashion to the chemical response-specific gene sets (see the next-gen TM-based gene set creation section). To validate the relevance of the these gene sets, we compared the top-25 gene sets obtained from the weighted global test to the effects of triazoles seen in rat postimplantation Whole Embryo Culture (WEC) [28]. The top-25 gene sets for embryonic structures resulting from the weighted global test correlated well with effects on the branchial arches, otic vesicles, posterior neuropore, heart, and somites as seen in the WEC (Table 4). Three gene sets could not be translated to changes seen in the WEC, since there is no scoring parameter for these changes in the WEC. Two of these gene sets concern the cloacal membrane. Even though there is no annotation for these in the WEC, the results correspond well with the in vivo data in the ToxRefDB_DevTox database (triazoles give rise to urogenital malformations). The third gene set (structure of embryo stage 6) has no direct correspondence in the WEC, but might be linked to the decrease in Total Morphological Score as seen in the WEC.

**Table 4:** The top 25 embryonic structure gene sets

**Embryonic structure**	***p*-value**	**WEC effect**
neural plate and or tube	1.5e-07	Posterior neuropore open
second branchial arch structure	4.9e-06	Branchial bars deformed
fourth branchial arch structure	4.9e-06	Branchial bars deformed
entire fourth branchial arch	4.9e-06	Branchial bars deformed
structure of first pharyngeal pouch	6.3e-06	Otic vesicles deformed
entire first pharyngeal pouch	6.3e-06	Otic vesicles deformed
entire neural tube	6.4e-06	Posterior neuropore open
Branchial Region	7.3e-06	Branchial bars deformed
entire second branchial arch	7.3e-06	Branchial bars deformed
structure of tympanic annulus	7.3e-06	Otic vesicles deformed
entire tympanic annulus	7.3e-06	Otic vesicles deformed
Neuroectoderm	7.5e-06	Posterior neuropore open
entire cloacal membrane	9.8e-06	*No corresponding scoring parameter available in the WEC*.
branchial arch structure	1.1e-05	Branchial bars deformed
structure of cloacal membrane	1.1e-05	*No corresponding scoring parameter available in the WEC*.
entire ostium secundum	1.1e-05	Heart ventrally turned
structure of third aortic arch	1.1e-05	Heart ventrally turned
entire branchial arch	2.2e-05	Branchial bars deformed
Otic Vesicle	2.2e-05	Otic vesicles deformed
entire auditory vesicle	2.2e-05	Otic vesicles deformed
structure of embryo stage 6	2.2e-05	*No corresponding scoring parameter available in the WEC*.
structure of early somite stage	2.2e-05	Somites irregular
entire early somite stage	2.2e-05	Somites irregular
third branchial arch structure	2.2e-05	Branchial bars deformed
entire third branchial arch	2.2e-05	Branchial bars deformed

False discovery rate-corrected *p*-values for the top 25 embryonic structure gene sets from the weighted global test on triazole gene expression data from the embryonic stem cell test, together with the effect seen in rat postimplantation Whole Embryo Culture (WEC).

**Figure 4:**
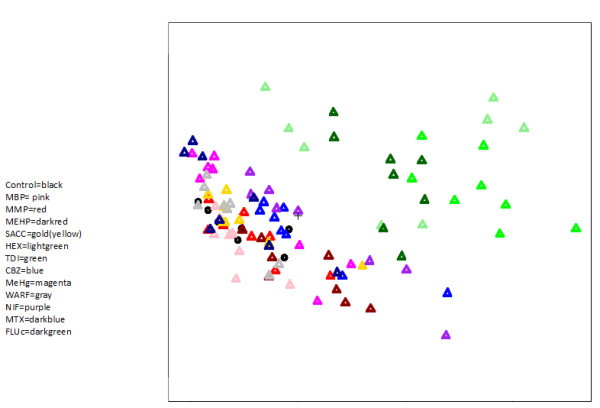
**Discrimination of triazoles using PCA. **Discrimination of triazoles (hexaconazole (HEX), triadimefon (TDI), flusilazole (FLUc)) from other developmentally toxic compounds (monobutyl phthalate (MBP), monoethylhexyl phthalate (MEHP), carbamazepine (CBZ), methylmercury (MeHg), warfarin (WARF), nitrofen (NIF), methotrexate (MTX)), non-toxic compunds (monomethyl phthalate (MMP), saccharine (SACC)) and time-matched unexposed cultures (Control) using principal component analysis on the basis of the leafnodes from the neural plate/tube gene set. Variance of the first principal component (horizontal axis): 70%. Variance of the second principal component (vertical axis): 14%.

In order to discriminate triazoles from other developmental and non-developmental toxicants using PCA, the most significant genes in the gene set with the lowest p-value from the weighted global test were used in the PCA analysis. These genes were extracted by calling the leafNodes function in the global test package. The leafNodes function gives an efficient summary of the test result by extracting the most significant subset of genes within a gene set using the inheritance multiple testing procedure of J.J. Goeman and L. Finos (as explained in the manual for the weighted global test). The "neural plate/tube" embryonic structure gene set had the highest FDR-corrected *p*-value (1.5e-07) of the 442 embryonic structure gene sets tested with the weighted global test against the triazoles gene expression data set. No embryonic structure gene sets came out significant after FDR correction for the saccharine gene expression data set. The "neural plate/tube" gene set contained 993 genes, and the 13 genes contributing most to the test (i.e. the leaf nodes) (Table 5) were used for PCA discrimination. Gene expression values of these selected genes were derived from the triazoles data set. These data were used to evaluate discrimination of triazoles from other developmental and non-developmental toxicants using PCA. Gene expression changes within these 13 genes indicated clear separation between the triazoles on one hand and the other compound-exposed cultures as well as the unexposed time-matched cultures on the other hand (Figure 4).

**Table 5:** The neural plate/tube gene set

**Entrez gene ID**	**Gene symbol**	***p*-value**
15394	HOXA1	5.2e-10
14472	GBX2	2.4e-07
64290	FOXB1	6.4e-05
94222	OLIG3	2.9e-04
218772	RARB	4.1e-05
20668	SOX13	1.5e-07
22413	WNT2	1.2e-05
320202	LEFTY2	1.5e-04
17292	MESP1	6.9e-05
14174	FGF3	1.1e-04
54352	IRX5	1.2e-05
18423	OTX1	3.1e-07
57028	PDXP	2.0e-06

False discovery rate-corrected *p*-values for the genes from the neural plate/tube gene set that contributed most to the weighted global test.

In short, 33 chemicals could be linked to the gene expression changes induced by triazoles. Toxicants corresponding to 21 of these 33 had a similar morphological developmental in vivo toxicity pattern as the triazoles. Using next-gen TM-derived gene sets for embryonic structures, we confirmed the relation between gene expression patterns and embryotoxic effects seen in the Whole Embryo Culture, and discriminated triazoles from other chemicals in a principal component analysis.

## Discussion

In the present study we perform identification of the chemical treatment, pharmacological mechanism elucidation, and compound toxicity profile comparison by testing chemical response-specific next-gen TM-based gene sets with GSA methods against different gene expression data sets. Such experiments have been performed before but were limited to evaluation of one specific tool and used a limited number of literature-based gene sets [3,7,9]. In contrast to the tool-specific gene sets that were used for identification of the chemical treatment in Minguez *et al.*, we provide the size and scope of our chemical response-specific gene sets, and make them available for download in a generic format (http://www.biosemantics.org/index.php?page=chemical-response-specific-gene-sets). We extend the evaluation of literature-based gene sets for identification of the chemical treatment performed by Patel and Butte to include next-gen TM. In doing this, we were able to create many more chemical response-specific gene sets (~30,200 gene sets for both human and mouse using next-gen TM compared to ~1,200 gene sets for human and ~600 gene sets for mouse using the CTD). While Jelier and co-workers tested next-gen TM-based gene sets for drugs on one gene expression data set using one GSA method, we tested next-gen TM-based gene sets on diverse sets of gene expression data with a variety of methods. No GSA method consistently outperformed the other, and we recommend using more than one GSA method when analysing gene expression data. We showed that gene sets generated by next-gen TM are less compound-specific than gene sets based on manual curation efforts but perform equal to or better than manual curation efforts in elucidating the pharmacological mechanism of fibrates. In addition, we show that next-gen TM allows for toxicity profile comparison of compounds for which manual annotation efforts are lacking (triazoles). We also successfully use next-gen TM-based gene sets for embryonic structures to describe effects induced by the triazoles hexaconazole, triadimefon and flusilazole in the EST, and to discriminate the triazoles from other chemicals using PCA.

A possible limitation of our approach is that we do not take the nature of the relation (for example expression (negative or positive) or phosphorylation) between the genes and chemicals or genes and embryonic structures into account when creating the gene sets. In the CTD, such information has been manually curated for the chemical-gene interactions. For the gene sets generated by text mining, such relations could be mined from the literature by using an ontology of known biological relations. Even though it might seem logical to only include curated expression relations, it also limits the possibility of finding new relations. From our omics point of view, all associations are of possible interest. In the future, it could be worth investigating how the nature of the relation influences the results of the GSA.

Another limitation relates to the number of gene expression data sets we used to test our methods on and the species variety (mouse and human). We choose these two species as a starting point since the annotation information available is more complete than for other genomes, but the amount of gene expression data and gene annotations for other model organisms in toxicology such as rat and zebrafish is increasing, and gene set creation and testing for these and other organisms is a topic for future research.

The amount of information in PubMed and CTD continue to increase. For example, following our “79983-71-4 OR hexaconazole” PubMed search example where we were able to retrieve 69 articles on November 21 (2011), the same search now (October 6 (2012)) retrieves 80 articles. A topic for future research would be to investigate how this information growth affects the gene set predictions.

Also, the process of updating the concept association scores is non-trivial. A conversion of the methods to a Web service environment, preferably with a Web interface, would greatly enhance its usability and is a project we are currently undertaking.

### Identification of chemical treatment

In this case study, we aimed to show that next-gen TM-based gene sets compare well to CTD-based gene sets in associating chemicals with gene expression data sets. The CTD-based gene sets ranked higher than the next-gen TM-based gene sets for all gene expression data sets except the ZnSO4 data set. This might be expected since the CTD-based gene sets are curated. However, t-profiling of the CTD-based and next-gen TM-based gene sets showed similar t-profiles for three of the six gene sets, indicating a similar gene set significance pattern for these gene sets despite the higher ranks of the CTD-based gene sets. The ranks for the CTD-based and next-gen TM-based gene sets differed between the different tools and between the experiments, indicating that in general, not one tool is to be preferred over the other when performing GSA analysis-based connectivity mapping. One needs to select the tool best suited for the experimental design. For example, ToxProfiler performs well with small number of replicates, while the weighted global test deals well with small changes in gene expression levels. The CTD-based gene sets had the highest significant ranks when using GeneCodis, which is in line with what Patel and Butte [3] reported for these experiments (using the hypergeometric test). The co-occurring annotations option in GeneCodis proved useful compared to the single annotation option in some cases.

### Pharmacological mechanism elucidation

In this case study, we aimed to associate PPARalpha agonists (fibrates) with a PPARalpha knock-out gene expression data set, and to show that the significantly scoring chemicals relate to relevant biological processes. The next-gen TM-based fibrate gene sets ranked similar or better than the CTD-based fibrate gene sets using two of the GSA tools, and all next-gen TM-based fibrate gene sets were significant using all GSA tools. Clearly, for this experiment, next-gen TM presents a comparable alternative to manual inspection of the literature. This might be the case since there is pharmacological and toxicological consensus on the action of peroxisome proliferators, and as such the literature accumulated over the past is more clearly described. The CTD-based gene sets for gemfibrozil and bezafibrate did not score significant using ToxProfiler and GeneCodis. These fibrates also had the lowest number of genes annotated to them: only four for gemfibrozil and nine for bezafibrate could be mapped to the current gene expression experiment. The size of the gene set has less influence when using the weighted global test compared to the other tools, which might explain that these gene sets scored significant using this method. The next-gen TM fibrate gene sets ranked much lower when using the weighted global test compared to the other tools. However, rankings were improved compared to the results by Jelier and coworkers [9]. The non-significant results for the fibrates when using cMap indicates that GSA tools present a good alternative when performing connectivity mapping.

We annotated the top-scoring chemicals for CTD and next-gen TM in Anni with biological processes to investigate if these corresponded to the ones annotated by hand in the PPARA study. For all GSA tools, biological processes corresponding to all categories reported in the PPARA study were found, except intestinal motility concepts. Intestinal motility concepts could be annotated to the significantly scoring signatures in cMap, while only one more (fatty acid oxidation) of the other five biological processes could be found using this method. These results show that the top scoring chemicals from the GSA analyses represent underlying biological processes relevant to the gene expression experiment, both for the CTD-based and next-gen TM-based gene sets.

### Compound toxicity profile comparison

The triazoles gene expression data set was analyzed with the aim to predict chemicals with a toxicity profile similar to triazoles and to discriminate triazoles from other developmental toxicants, and non-toxic compounds. Many (64%) of the predicted chemicals had an in vivo toxicity pattern corresponding to that of the triazoles. The highest ranking compound with a toxicology pattern different from the triazoles according to ToxRefDB_DevTox was Monosodium methane. Three of the eight genes that contributed most to the significance of this compound where annotated with the concept “axial skeletal structure” in Anni. Triazoles cause malformations in this structure according to the ToxRefDB_DevTox. Even though Monosodium methane does not cause malformations in the axial skeletal structure of rats or rabbits according to the ToxRefDB_DevTox, our results suggest that the compound should be tested for in vivo toxicity also in mouse. On the level of risk assessment we are interested in the situation in man, and the more information about the toxic effects of compounds in different systems the better. The highest ranking non-toxic compound (Zoxamide) scored significant mainly because of the presence of the gene CYP51A1 in the gene set. The fungicidal mode of action of triazoles is based on the inhibition of this gene [29]. We noted five significantly scoring chemicals for the saccharine gene expression experiment of which four were developmental toxicants, which confirms that gene set testing with chemical response-specific gene sets should be considered as hypothesis generation and that every significantly scoring compound need to be investigated further. This is something that also applies to standard connectivity mapping. Using the weighted global test, further investigation would constitute inspection of the leaf nodes in the gene sets, since these genes contribute most to the test. Anni can be used to infer the function of the leaf nodes, give information on how these are connected to each other, and via direct links to the literature more thorough information can easily be found.

De Jong and co-workers [28] showed that the embryonic stem cell test (EST) is able to give a relatively good potency ranking compared with the in vivo developmental toxicity potency of triazoles, but pointed out that the system gives little information on the type of effects that can occur after exposure to the chemicals. We show that by associating embryonic structure gene sets with a triazole gene expression profile obtained through the EST, effect information becomes readily available. Also, the genes in the embryonic structure gene set that contributed most to the weighted global test could separate triazoles from other chemicals in a PCA. Genes that effectively can separate between chemical classes are usually searched for by applying a statistical test on differential gene expression (see for example [30]). Here, we show that such genes can also be found by applying the weighted global test with relevant gene sets (in this case embryonic structure gene sets).

## Conclusions

In conclusion, GSA with next-gen TM-derived chemical response-specific gene sets is a scalable method for identifying similarities in gene responses to other chemicals, from which one may infer potential mode of action and/or toxic effect.

## Competing interests

The authors declare that they have no competing interests.

## Authors’ contributions

KMH conceived of the study, participated in its design and coordination, created the gene sets, carried out all bioinformatic analyses except the ones with ToxProfiler, and drafted the manuscript. AB participated in the design of the study and carried out the bioinformatic analyses with ToxProfiler. DAMD participated in the design of the study and carried out the principal component analysis. JJG participated in the statistical and bioinformatic analyses and helped to draft the manuscript. EJ compared the results from the gene sets analysis to the effects of triazoles seen in rat postimplantation Whole Embryo Culture. AHP participated in the design of the study and helped to draft the manuscript. RHS participated in the design of the study and helped to draft the manuscript. JCK participated in the design of the study and helped to draft the manuscript. JAK participated in the design and coordination of the study, and helped to draft the manuscript. All authors read and approved the final manuscript.

## Pre-publication history

The pre-publication history for this paper can be accessed here:

http://www.biomedcentral.com/1755-8794/6/2/prepub
